# Investigation of Time Series Representations and Similarity Measures for Structural Damage Pattern Recognition

**DOI:** 10.1155/2013/248349

**Published:** 2013-09-26

**Authors:** Wenjia Liu, Bo Chen, R. Andrew Swartz

**Affiliations:** ^1^Elektrobit Automotive Inc. Detroit, Farmington Hills, MI 48331, USA; ^2^Department of Mechanical Engineering—Engineering Mechanics, Department of Electrical and Computer Engineering, Michigan Technological University, Houghton, MI 49931, USA; ^3^Department of Civil and Environmental Engineering, Department of Electrical and Computer Engineering, Michigan Technological University, Houghton, MI 49931, USA

## Abstract

This paper investigates the time series representation methods and similarity measures for sensor data feature extraction and structural damage pattern recognition. Both model-based time series representation and dimensionality reduction methods are studied to compare the effectiveness of feature extraction for damage pattern recognition. The evaluation of feature extraction methods is performed by examining the separation of feature vectors among different damage patterns and the pattern recognition success rate. In addition, the impact of similarity measures on the pattern recognition success rate and the metrics for damage localization are also investigated. The test data used in this study are from the System Identification to Monitor Civil Engineering Structures (SIMCES) Z24 Bridge damage detection tests, a rigorous instrumentation campaign that recorded the dynamic performance of a concrete box-girder bridge under progressively increasing damage scenarios. A number of progressive damage test case datasets and damage test data with different damage modalities are used. The simulation results show that both time series representation methods and similarity measures have significant impact on the pattern recognition success rate.

## 1. Introduction

Time series is one of the most commonly used data formats in real world. It is being generated in a tremendous speed from almost every application area. Processing raw time series data is expensive due to its high dimension. Two key aspects for achieving effectiveness and efficiency when managing time series data are representation methods and similarity measures [1]. In the last decades, a number of representation methods and similarity measures have been proposed to extract features from time series data for indexing, classification, and clustering. The objective of feature extraction is to find a representation at a lower dimensionality that preserves the fundamental characteristics of the original time-series data [2]. The time series representation methods can be classified as shape-based method, structure-based (or model-based) method, and dimensionality reduction. For long time series data, model-based and dimensionality reduction methods are more effective. 

Model-based time series representation methods extract global features from time series, create feature vectors, and use these feature vectors to measure similarity of time series for classification and clustering. Time series data are usually fitted into models, such as Box Jenkins model or Markov Model, and the parameters of the model are used to form feature vectors. The dimensionality reduction methods are typically based on data transformation. Many dimensionality reduction methods have been reported in the literature, such as discrete Fourier transformation (DFT) [3, 4], single value decomposition (SVD) [5], discrete wavelet transformation (DWT) [6], piecewise approximation [7], and Chebyshev polynomials (CHEB) [8]. 

Similarity measure is important for both evaluating feature extraction methods and time series classification. Feature extraction process consists of following steps: establishing a distance metric, producing a dimensionality reduction technique that reduces the dimensionality of the data from *n* to *N* (where *N* < *n*), and producing a distance measure defined on the *N*-dimensional representation of the data. There are over a dozen distance measures that have been reported in the literature for mining and indexing time series. These similarity measures include Euclidean distance [4], Mahalanobis distance, Cosine distance, Standardized Euclidean (Seuclidean) distance, Correlation distance, and Dynamic Time Warping (DTW) [9, 10]. 

This paper examines several time series representation methods and similarity measures for structural damage feature extraction and pattern recognition. Smart sensors have been widely used for structural health monitoring, and sensor data-based structural damage detection has received increased attention recently [11–13]. In this paper, pattern-recognition-based structural damage detection and classification are based on the similarity measure of damage feature vectors with normal feature vectors. The goal of the feature extraction is to select features which will result in the separation of damage feature vectors and normal feature vectors in the feature space. This will allow us to distinguish damage and normal patterns. The performance of representation methods and similarity measures are evaluated utilizing acceleration data collected from the Z24 Bridge as part of the System Identification to Monitor Civil Engineering Structures (SIMCES) project.

The rest of the paper is organized as follows.  Section 2 introduces the Z24 bridge datasets which are used for the validation.  Section 3 presents feature representation methods studied in this paper for structural damage feature extraction from time series sensor data.  Section 4 evaluates the effects of multiple similarity measures and the length of time series data on the performance of structural damage pattern recognition.  Section 5 concludes the work. 

## 2. Validation Structural Data: Z24 Bridge Datasets

To investigate the effectiveness of time series representation methods and similarity measures applied to structural damage pattern recognition, the Z24 Bridge test datasets are used as validation data in this paper [14]. The Z24 Bridge datasets are especially appealing because the progressive damage scenarios include scenarios of the same type of damage but varying levels (support settlement) as well as radically distinct damage modalities (support settlement versus concrete spalling versus damage to pretension elements). These features of the damage scenarios allow us to differentiate between damage patterns that differ based on damage modality versus damage patterns that differ based on damage severity.

Sensors collecting global level vibrational data (e.g., displacements or accelerations in frequency ranges consistent with global modes of the structure) are capable of capturing dynamic effects that can give an indication of the overall health of the structure. The Z24 Bridge datasets are global level vibrational data that are well known within the civil structural health monitoring community and that have been made widely available for other health monitoring studies. The SIMCES project began in 1997 with a goal to collect real-world data from an operational bridge under realistic damage scenarios. The Z24 Bridge, crossing Bern to Zurich highway and located between Koppigen and Utzenstorf, Switzerland, was heavily instrumented and tested under a systematic program of progressive damage scenarios before it was demolished to make way for a new railway line [14]. Extensive acceleration measurements were made both from the undamaged bridge (correlated with environmental effects) and during the progressive damage scenarios. Data from this project has been used in a number of published studies on the properties of the structure [15–20] as well as damage detection strategies [20–25]. 

**Figure 1:**
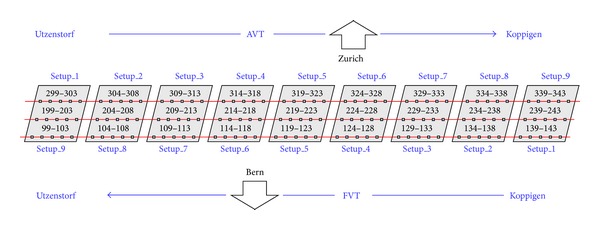
Measurement setup for vibration test on Z24 bridge [26].

The bridge itself was a three-span, medium-span prestressed concrete, two-cell, closed box-girder bridge with concrete columns. Global level acceleration data were recorded in both ambient vibration test (AVT) and forced vibration test (FVT). Two vertical shakers were used to excite the bridge for the forced tests. The distribution of bridge surface accelerometers is shown in Figure 1. A series of progressive damage cases were applied beginning with the most reversible cases (including multiple levels of support settlement) and progressing to irreversible cases (e.g., concrete spalling, damage to prestressing tendons, anchor heads, etc.).  Table 1 provides a list of damage scenarios. In the presented study, the data collected from these damage scenarios are divided into training and test subsets. The training subset was used to generate representative feature vectors for damage patterns, and the test subset was used to find the success rate of the pattern recognition.

## 3. Feature Representation of Time Series Sensor Data 

**Figure 2:**
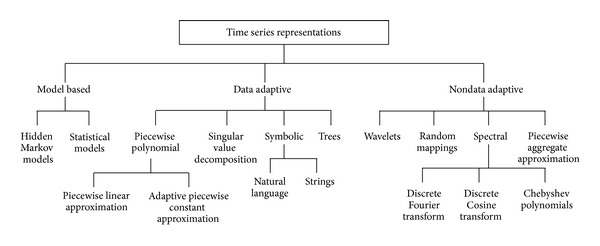
Time series representations [27].

Many high-level representations of time series data have been proposed for similarity search and data mining as shown in Figure 2, including single value decomposition (SVD) [5], discrete Fourier transformation [3, 4], discrete wavelet transformation [6], adaptive piecewise constant approximation [7], discrete cosine transformation [5], Chebyshev polynomials [8], piecewise aggregate approximation [29], and symbolic aggregate approximation [30]. In this paper, autoregressive (AR) model-based and dimensionality reduction (DFT and DWT) feature extraction methods are investigated.

### 3.1. Model-Based Feature Extraction Methods

In this paper, autoregressive model is used to model a time series sensor data. The AR model-based feature extraction method fits time series into an AR model and uses the coefficients of the AR model as members of the feature vector. For a time series sensor data *x*, it can be fitted into an AR model of order *p* as shown by

(1) $$\begin{array}{c} x_{k}=\sum_{i=1}^{p}\alpha_{i}x_{k-i}+r_{k},\;k=p+1,\ldots ,n, \end{array}$$

Where *α*_*i*_, *i* = 1,2,…, and *p* are the coefficients of the AR model. The order of AR model is 20 in this paper. The feature vector of the time series sensor data *x*, *F*(*X*), is formed by the coefficients of the AR model as shown by

(2) $$\begin{array}{c} F\left(X\right)={\left(\alpha_{1},\alpha_{2},\ldots ,\alpha_{P}\right)}^{T}. \end{array}$$

To reduce noise effects, the measurement sensor data *Z* are standardized by

(3) $$\begin{array}{c} x_{i}=\frac{z_{i}-\mu_{i}}{\sigma_{i}},\;i=1,2,\ldots ,n, \end{array}$$

where *μ*_*i*_ and *σ*_*i*_ are the mean and standard deviation of the time series *Z*.

### 3.2. Dimensionality Reduction Methods

#### 3.2.1. Discrete Fourier Transform

The Discrete Fourier Transform (DFT) is one type of discrete transforms which transforms a function in the time domain into another in the frequency domain. Given a time series *x* with the length of *n*, the DFT of *x* is defined to be *X* consisting of *n* complex numbers *X*_*k*_, *k* = 1,2,…*n* as shown by

(4) $$\begin{array}{c} X_{k}=\sum_{i=1}^{n}{x_{i}e}^{-j(2\pi /n)(k-1)(i-1)},\;k=1,2,\ldots n. \end{array}$$

To perform the dimensionality reduction of the time series *X* into a reduced feature space of dimensionality *R*, two feature selection methods are compared. The first method uses the first *R* number of DFT coefficients to form an *R*-dimensional feature vector to represent the time series *X* in the *R*-dimensional feature space [4]. The second method uses first 8 model frequencies and corresponding signal amplitudes to form feature vectors. Model frequencies of the bridge presented in [31] are used as references in the search of real modal frequencies and signal amplitudes in sensor time series. Assume that the first 8 model frequencies and amplitudes of a time series signal are *f*_1_, *f*_2_, *f*_3_, *f*_4_, *f*_5_, *f*_6_, *f*_7_, and *f*_8_ and *a*_1_, *a*_2_, *a*_3_, *a*_4_, *a*_5_, *a*_6_, *a*_7_, and *a*_8_, the feature vector of the time series is defined as

(5) F(X)=(f1,k×a1,f2,k×a2,f3,k×a3,f4,k× a4, f5,k×a5,f6,k×a6,f7,k×a7,f8,k×a8),

where *k* is the weight factor of the amplitudes. 

#### 3.2.2. Discrete Wavelet Transform

Discrete wavelet transform decomposes a signal into layers of coefficients. These coefficients contain both frequency and time domain information. Discrete wavelet transform has been applied for feature extraction in different fields [32–34]. Given a time series *x* with the length of *n*, the discrete wavelet transform (DWT) of *x* is calculated by passing the time series signal through a series of low pass and high pass filters as shown by

(6) $$\begin{array}{c} y_{l}\left[n\right]=x\left[n\right]*g\left[n\right]=\sum_{k=-\infty }^{\infty }x\left[k\right]g\left[n-k\right],\\ y_{h}\left[n\right]=x\left[n\right]*h\left[n\right]=\sum_{k=-\infty }^{\infty }x\left[k\right]h\left[n-k\right], \end{array}$$

where *g*[*n*] and *h*[*n*] are low pass filter and high pass filter, respectively. The outputs of the high pass filter are detail coefficients, while the outputs of the low pass filter are approximation coefficients. The approximation coefficients are further decomposed in the next iteration while the detail coefficients are kept as the current level wavelet coefficients. 

To form feature vectors from wavelet coefficients, feature extraction method proposed in [28] is employed. This feature extraction method consists of two steps: cluster determination and feature extraction. The cluster determination process divides the wavelet coefficients into a number of clusters *c*_1_, *c*_2_,…*c*_*k*_, and the feature extraction process calculates the feature vector for a time series of sensor data. The elements of a feature vector are Euclidean norms of each cluster *F* = (||*c*_1_||_2_, ||*c*_2_||_2_,…||*c*_*k*_||_2_). The clusters *c*_1_, *c*_2_,…*c*_*k*_ are determined as row vectors such that each cluster contains a significant wavelet coefficient near the midpoint of each cluster. 

**Figure 3:**
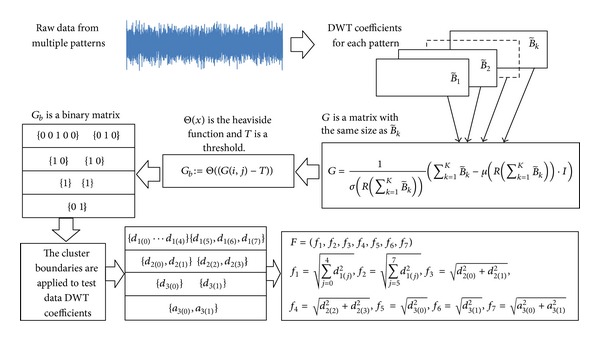
Feature extraction from DWT coefficients [28].

Figure 3 shows the process of cluster determination and feature extraction from the sensor data of multiple data patterns. First, the DWT coefficient matrices of sensor data from multiple patterns are calculated. The dimensions of these coefficient matrices are the same if time series sensor data have the same length. To find significant wavelet coefficients, the Central Limit Theorem [35] is applied to the elements of the DWT coefficient matrices to generate a new matrix *G* as shown by

(7) $$\begin{array}{c} G=\frac{1}{\sigma \left(R\left(\sum_{k=1}^{K}{\tilde{B}}_{k}\right)\right)}\left(\sum_{k=1}^{K}{\tilde{B}}_{k}-\mu \left(R\left(\sum_{k=1}^{K}{\tilde{B}}_{k}\right)\right)\cdot I\right), \end{array}$$

where *R* is the operator to reduce a matrix by its last row and *I* is a matrix which has the same size as ${\tilde{B}}_{k}$ and has all the elements of 1. The members of the *G* matrix are then compared with a threshold and save the comparison results to the corresponding location in a matrix *G*_*b*_. The comparison result is 1 when the member of the *G* matrix is greater than the threshold and 0 when the member of the *G* matrix is less than the threshold. Pittner and Kamarthi [28] prove that the 1s in the matrix *G*_*b*_ occur at the same locations where the significant wavelet coefficients occur in the matrices ${\tilde{B}}_{k}$. Based on the *G*_*b*_ matrix, the clusters are then formed with the following rules: (1) each cluster contains one “1” element and (2) if one row contains no “1” element, this row is treated as one cluster. After the boundaries of each cluster are determined from the *G*_*b*_ matrix, the wavelet coefficients in the ${\tilde{B}}_{k}$ matrices are grouped into clusters using the cluster boundary information obtained from the *G*_*b*_ matrix. The feature vector of the ${\tilde{B}}_{k}$ matrix is calculated with the Euclidean norms of each cluster as shown in Figure 3.

## 4. Performance Evaluation

Performance evaluation was conducted to test the effectiveness of the feature extraction methods. Two test scenarios were designed: (1) same type of structural damage with different extents (patterns 2–6 in Table 1) and (2) different damage modalities (patterns 6, 10, 11, 12, 14, and 16 in Table 1). We adopted sensor data collected by sensor node 232 in the forced vibration test. Data points in the sensor data files were divided into two groups: training data and test data. Feature vectors generated from training data were used to find the representative feature vectors for each damage pattern using *K*-means method. The feature vectors created from test data were used to test the effectiveness of feature extraction methods for damage pattern recognition using *K*-nearest neighbor (*K*NN-1) classification method.

To find good similarity measures for structural damage pattern recognition, a number of commonly used similarity measures are evaluated using Z24 bridge datasets. The tested similarity measures include Manhattan distance, Euclidean distance, L-infinity (Maximum) norm, Mahalanobis distance, cosine distance, standardized Euclidean (Seuclidean) distance, and correlation distance. Let *X* and *Y* be two feature vectors with dimension *n*. The definitions of these similarity measures are given as follows.

(i) Manhattan distance:
  (8) $$\begin{array}{c} d_{XY}=\sum_{i=1}^{n}\left|X_{i}-Y_{i}\right|. \end{array}$$
(ii) Euclidean distance:
  (9) $$\begin{array}{c} D=\sqrt{\left(X-Y\right){\left(X-Y\right)}^{T}}. \end{array}$$
(iii) L-infinity:
  (10) $$\begin{array}{c} d_{XY}=\max\left(\left|X_{i}-Y_{i}\right|\right),\;i\in n. \end{array}$$
(iv) The Mahalanobis distance of a multivariate vector *X* = (*x*_1_, *x*_2_,…, *x*_*n*_)^*T*^ from a group of values with mean  *μ* = (*μ*_1_, *μ*_2_,…, *μ*_*n*_)^*T*^ and covariance matrix *S* is defined as
  (11) $$\begin{array}{c} D=\sqrt{{\left(X-\mu \right)}^{T}S^{-1}\left(X-\mu \right)}. \end{array}$$
(v) Cosine distance:
  (12) $$\begin{array}{c} d_{XY}=1-\frac{XY^{T}}{{\left(XX^{T}\right)}^{1/2}{\left(YY^{T}\right)}^{1/2}}. \end{array}$$
(vi) Standardized Euclidean (Seuclidean) distance:
  (13) $$\begin{array}{c} d_{XY}^{2}=\left(X-Y\right)D^{-1}{\left(X-Y\right)}^{T}, \end{array}$$
 where *D* is a diagonal matrix with diagonal elements given by *v*_*j*_^2^, which denotes the variance of the *j*th-feature over all the features vectors contained by *X* and *Y*.
(vii) Correlation distance:
  (14) $$\begin{array}{c} d_{XY}=1-\frac{\left(X-\bar{X}\right){\left(Y-\bar{Y}\right)}^{T}}{{\left(\left(X-\bar{X}\right){\left(X-\bar{X}\right)}^{T}\right)}^{1/2}{\left(\left(Y-\bar{Y}\right){\left(Y-\bar{Y}\right)}^{T}\right)}^{1/2}}, \end{array}$$

where $\bar{X}=\left(1/p\right)\sum_{j}X_{j},\bar{Y}=\left(1/p\right)\sum_{j}Y_{j}.$

### 4.1. The Effects of Similarity Measures and the Length of Time Series on the Performance of Pattern Recognition Using AR-Based Feature Extraction

To test the performance of feature extraction methods, the Z24 Bridge datasets described in Section 2 are used. In the Z24 bridge datasets, each sensor data file contains 65536 acceleration data points. To avoid unstable measurement data in the beginning of each test, the first 4999 data points are abandoned. The rest of the measurement data in data files are used for feature extraction. The first time series starts from 5000th data point. The next time series is formed by shifting 100 data points from previous time series. For example, the second time series starts from 5100th data point and the third time series starts from 5200th data point. Various lengths of time series are formed to test the impact of time series length on the performance of pattern recognition. The selected time series lengths include 100, 200, 300, 500, 700, 1000, 1500, 2000, 3000, and 5000. 

**Figure 4:**
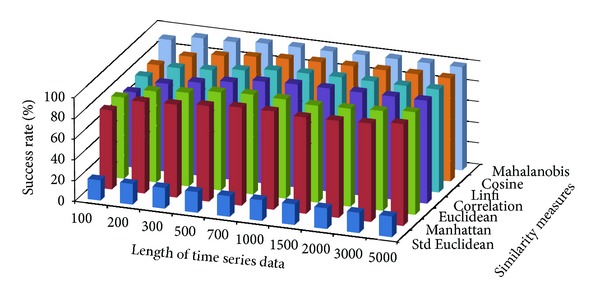
Average success rate of pattern recognition with different similarity measures and the length of time series in first scenario.

The success rate of classifying test data to corresponding damage patterns using AR-based feature extraction method was evaluated for different damage modalities and progressive damage patterns.  Figure 4 shows the average success rate of pattern recognition in first scenario (pattern 2–6 in Table 1) using similarity measures defined above. Five data patterns defined in the first scenario are No damage, pier 3 settlement—20 mm, pier 3 settlement—40 mm, pier 3 settlement—80 mm, and pier 3 settlement—95 mm. The *x*-axis stands for the length of time series for feature extraction; the *y* axis stands for the type of similarity measures; and the *z* axis is the average success rate of AR-based feature extraction method. From Figure 4, we can see that the Mahalanobis distance outperforms over other similarity measures. For each similarity measure, the success rate increases as the length of time series gets longer.

**Figure 5:**
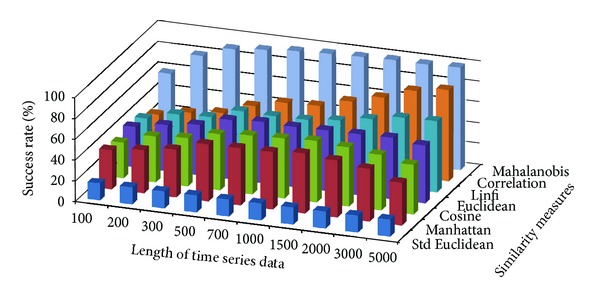
Average success rate of pattern recognition with different similarity measures and the length of time series in second scenario.

**Figure 6:**
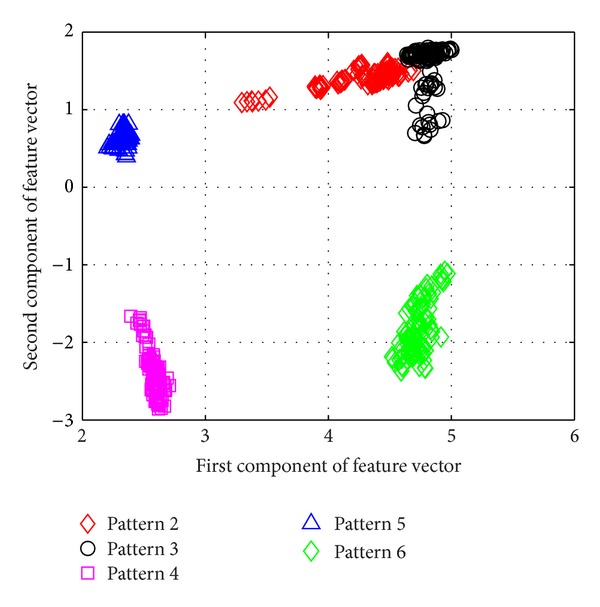
The AR feature vectors of data patterns in first scenario from sensor node 232 using Mahalanobis distance.

**Figure 7:**
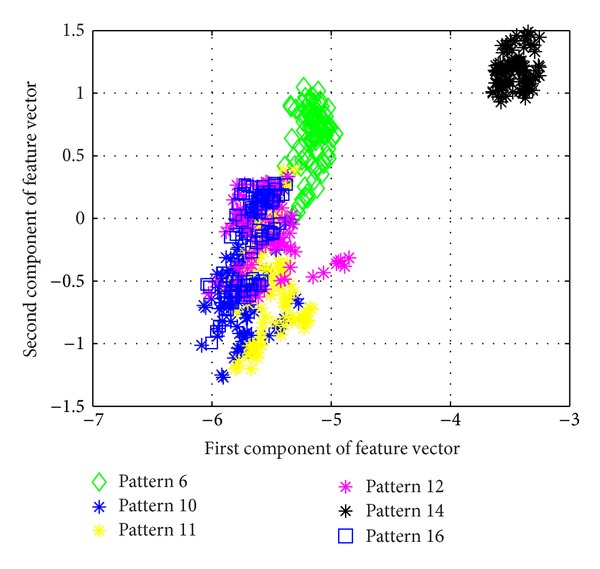
The AR feature vectors of data patterns in second scenario from sensor node 232 using Mahalanobis distance.

Figure 5 shows the average success rate of pattern recognition for Patterns 6, 10, 11, 12, 14, and 16 in Table 1 (pier 3 settlement—95 mm, concrete spalling—24 m^2^, landslide at abutment, concrete hinge failure, anchor head failure (4), and tendon wire failure (100/4)). Figure 5 presents similar trends as Figure 4 with regard to the effects of similarity measures on the pattern recognition success rate for AR-based feature extraction method. The success rate in Figure 5, however, is generally lower than in Figure 4. This is due to the separation of feature vectors in first scenario is better than that of the second scenario. This can be observed from Figures 6 and 7. These two figures show the distribution of AR feature vectors of data patterns in the first and second scenarios from the sensor node 232 using Mahalanobis distance. To display high dimensional feature vectors in 2D space, 20-dimensional feature vectors are reduced to 2-dimensional feature vectors using principal component analysis (PCA). The *x*-axis is the first component after PCA and the *y*-axis is the second component after PCA.

**Figure 8:**
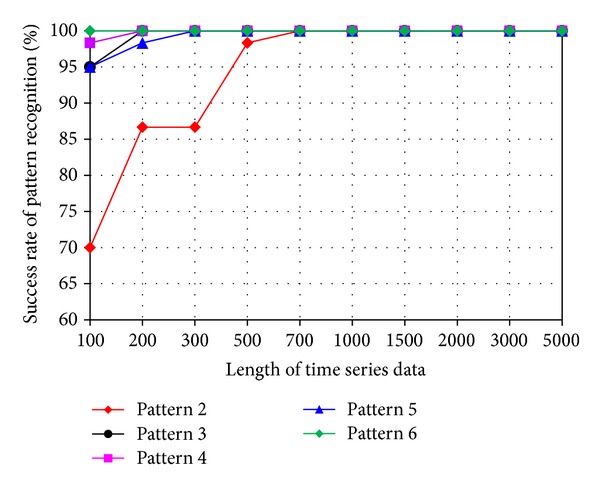
Success rate of pattern recognition for first scenario (pattern 2–6) using Mahalanobis distance.

**Figure 9:**
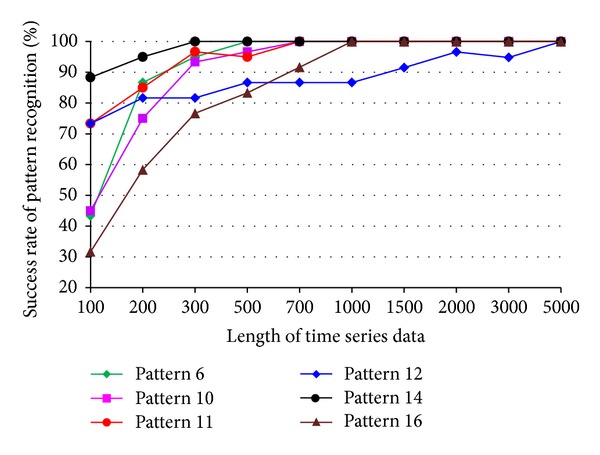
Success rate of pattern recognition for second scenario (patterns 6, 10, 11, 12, 14, and 16) using Mahalanobis distance.

The impact of the length of time series on pattern recognition success rate using Mahalanobis distance as similarity measure was also investigated.  Figure 8 shows the success rates of pattern recognition in first scenario and Figure 9 shows the success rates of pattern recognition in second scenario.  Figure 8 indicates that the success rate increases as the length of time series increases. In addition, the severity of damage affects the pattern recognition success rate. Pattern 6, with largest settlement, has the highest pattern recognition success rate. Figure 9 shows the success rate of pattern recognition performed on different damage modalities. Similarly, the success rates go up as the lengths of time series increases.

The success rate is also affected by the separation of feature vectors in feature space. Figures 6 and 7 show the distribution of feature vectors in the first and second scenarios. The length of time series is 5000 in both plots. In Figure 6, the feature vectors of pattern 6 are located far away from feature vectors of other patterns. As a result, pattern 6 is easy to be recognized. The success rate of pattern 6 is the highest one compared with other patterns.  Figure 7 shows the distribution of the feature vectors from different damage modalities. As we can see from Figure 7, feature vectors of pattern 14 are located far away from feature vectors of other patterns, so the success rates of pattern 14 is higher than the success rate of other patterns. In general, the separation of feature vectors in first scenario is better than that of the second scenario. The overall success rate in first scenario is also higher than that of the second scenario.

### 4.2. The Effects of Similarity Measures on the Performance of Pattern Recognition Using DFT-Based Feature Extraction

**Figure 10:**
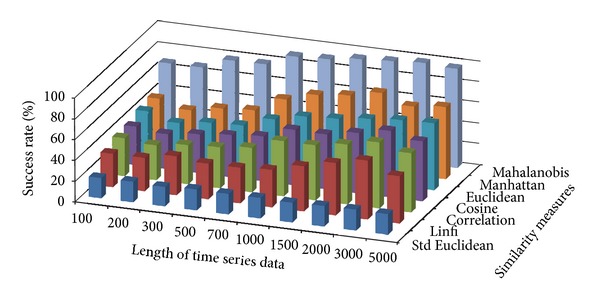
Average success rate of pattern recognition using DFT-based feature extraction in first scenario.

**Figure 11:**
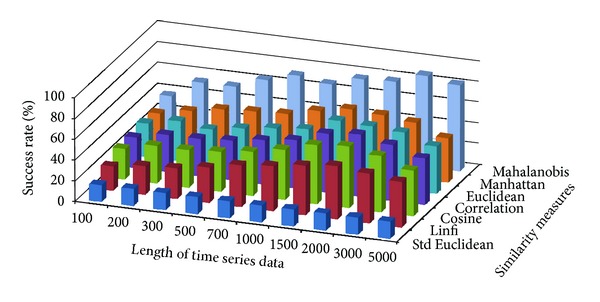
Average success rate of pattern recognition using DFT-based feature extraction in second scenario.

Figures 10 and 11 show the average success rate of DFT-based feature extraction method for structural damage pattern recognition with different similarity measures. In general, the success rate of DFT-based feature extraction is lower than that of AR-based feature extraction method. Compare with two test scenarios, the first scenario has relatively high success rate. In both test scenarios, the dissimilarity measure—Mahalanobis distance again showing better performance than other similarity measures.

### 4.3. The Effects of Time Series Length on Success Rate Using DFT-Based Feature Extraction

**Figure 12:**
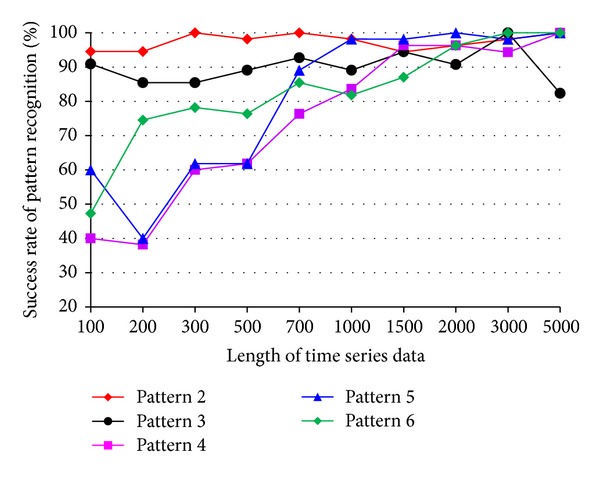
Success rate of pattern recognition using DFT-based feature extraction in first scenario.

**Figure 13:**
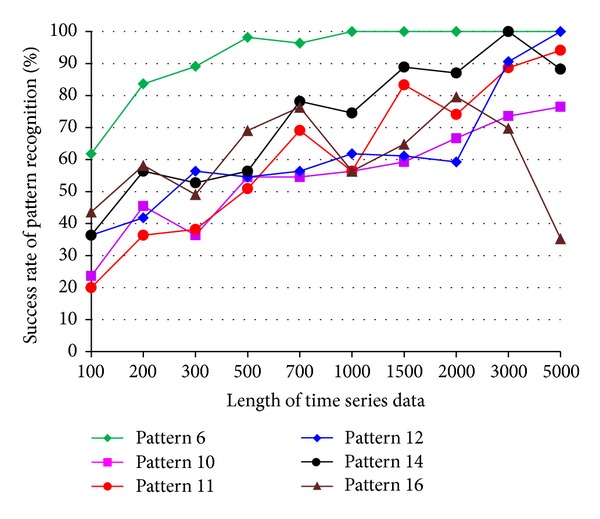
Success rate of pattern recognition using DFT-based feature extraction in second scenario.

**Figure 14:**
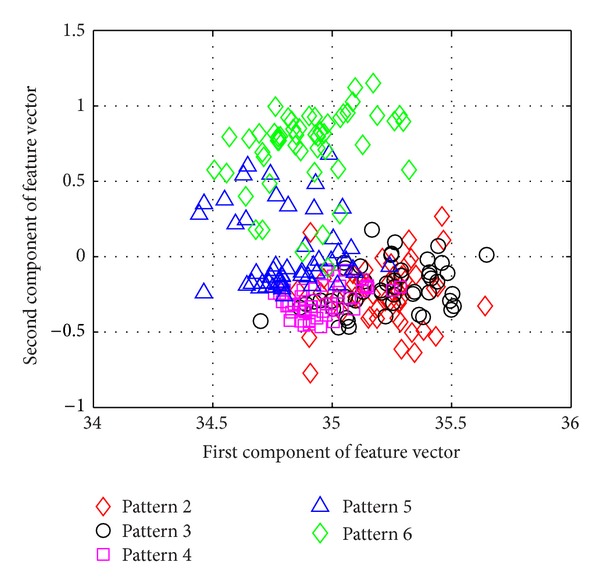
The DFT feature vectors of data patterns in first scenario from sensor node 232.

**Figure 15:**
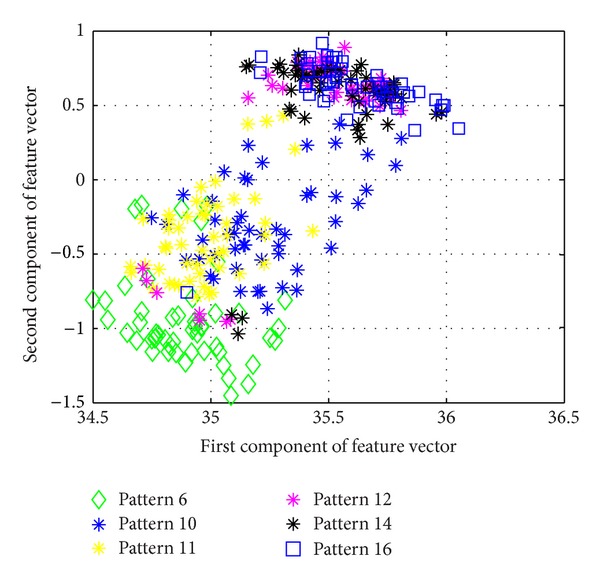
The DFT feature vectors of data patterns in second scenario from sensor node 232.

Figures 12 and 13 show the success rates of pattern recognition for each damage pattern with different lengths of time series. The similarity measure used in the tests is the Mahalanobis distance. For most damage patterns, the success rate increases as the length of time series increases. In general, the success rate of pattern recognition in first scenario is better than that of the second scenario. Figures 14 and 15 show the distribution of the feature vectors in two scenarios using DFT-based feature extraction method. The length of time series is 5000 in both plots. The separation of feature vectors using AR-based feature extraction method (Figures 6 and 7) is better than that of the DFT-based feature extraction method (Figures 14 and 15). As a result, the success rates of pattern recognition using AR-based feature extraction (Figures 8 and 9) are higher than that of the DFT-based feature extraction (Figures 12 and 13).

### 4.4. The Effects of Similarity Measures on the Performance of Pattern Recognition Using DWT-Based Feature Extraction

**Figure 16:**
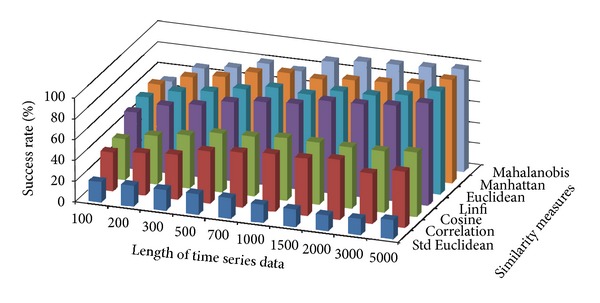
Average success rate of pattern recognition using DWT-based feature extraction in first scenario.

**Figure 17:**
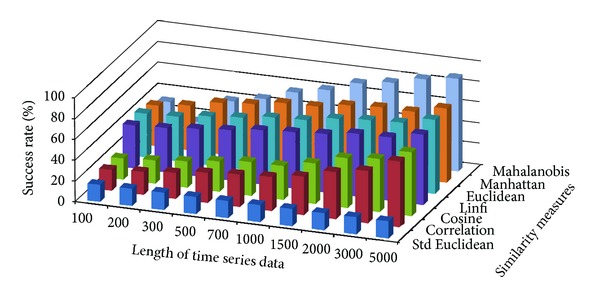
Average success rate of pattern recognition using DWT-based feature extraction in second scenario.

Figures 16 and 17 show the average success rate of DWT-based feature extraction method for structural damage pattern recognition with different similarity measures. In general, the success rate of DWT-based feature extraction is lower than that of AR-based feature extraction method but higher than that of the DFT-based feature extraction method. Compare with two test scenarios, the first scenario has relatively high success rate. In both test scenarios, the dissimilarity measure—Mahalanobis distance again showing better performance than other similarity measures.

### 4.5. The Effects of Time Series Length on Success Rate Using DWT-Based Feature Extraction

**Figure 18:**
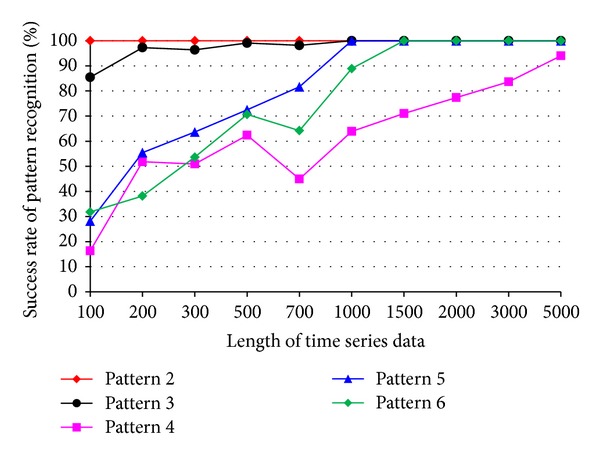
Success rate of pattern recognition using DWT-based feature extraction in first scenario (sensor 232).

**Figure 19:**
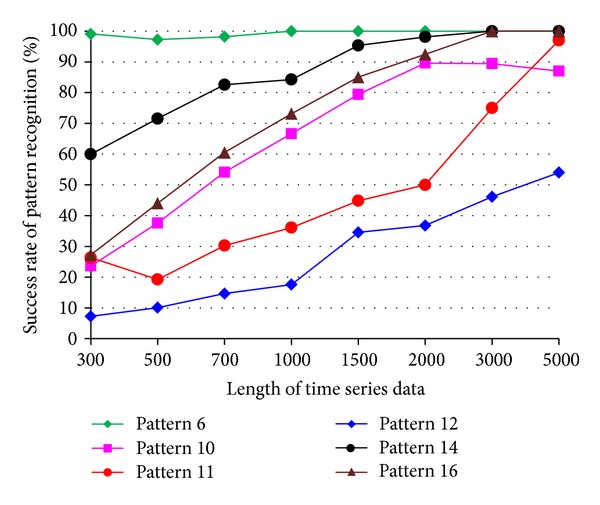
Success rate of pattern recognition using DWT-based feature extraction in second scenario (sensor 232).

**Figure 20:**
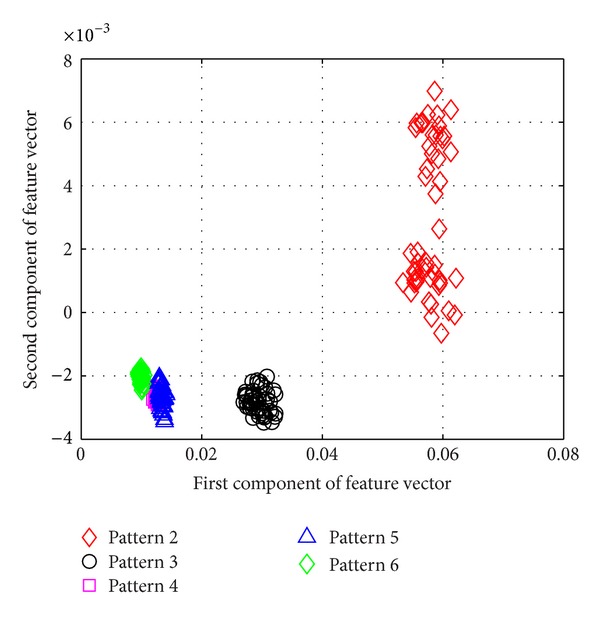
The DWT feature vectors of data patterns in first scenario from sensor node 232.

**Figure 21:**
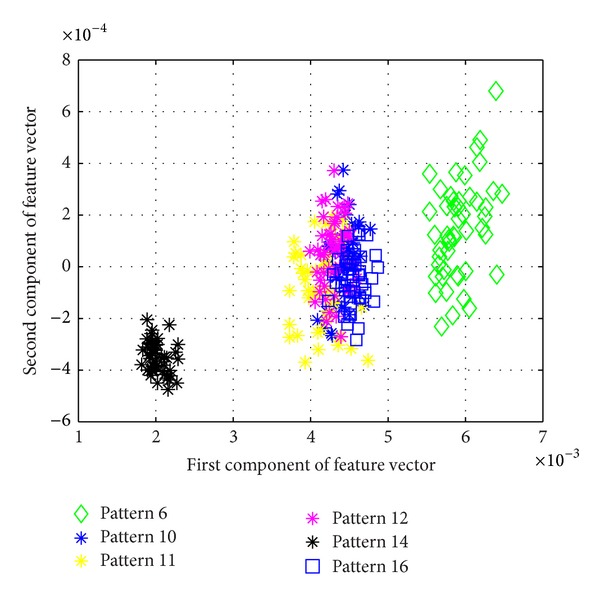
The DWT feature vectors of data patterns in second scenario from sensor node 232.

Figures 18 and 19 show the success rates of pattern recognition for each damage pattern with different lengths of time series. The similarity measure used in the tests is the Mahalanobis distance. For most damage patterns, the success rate increases as the length of time series increases. Figures 20 and 21 show the distribution of the feature vectors in two scenarios. The length of time series is 5000 in both plots. Comparing the success rate plot with feature distribution in both test scenarios, the impact of the separation of feature vectors on the success rate can clearly be seen again. The success rates of patterns 2 and 3 in the first scenario are much higher compared with other patterns, and the success rates of patterns 6 and 14 in the second scenario are much higher compared with other patterns. 

### 4.6. Damage Localization Analysis Using Pattern Recognition Approach

Damage localization is important when the damage is detected. To investigate the applicability of pattern recognition approach for structural damage localization, the numerical analysis study has been conducted to examine the shift of the representative feature vectors of the damage patterns from the normal pattern in the feature space using damage pattern 6 data of Z24 Bridge. To find out the potential relationship between damage location and the feature vectors of sensor data, the distances between normal pattern feature vectors and damage pattern 6 feature vectors are calculated. The similarity measure used in the calculation is Mahalanobis distance as

(15) Feature  shift  distance  =Mahalanobis(damage  feature  vectors,            normal  feature  vectors).

**Figure 22:**
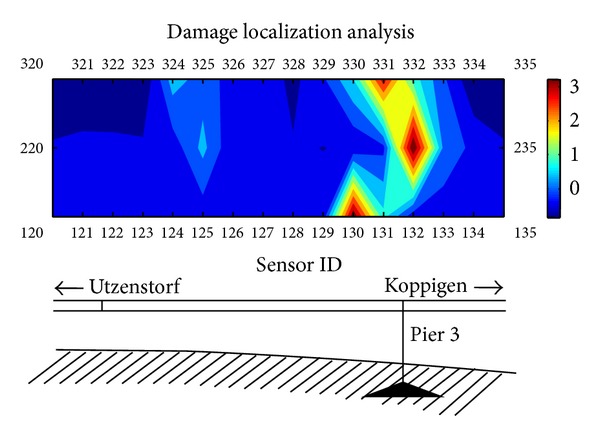
The shifted distances of damage pattern 6 feature vectors from normal pattern feature vectors.

Figure 22 shows the feature vector shift of damage pattern 6 from the normal pattern on the sensor nodes 120–320. The distribution of these sensor nodes on the bridge is indicated by numbers corresponding to their IDs as shown in Figure 22. There are three rows of sensor nodes. The sensor nodes 120–135 form the first row and are located in the front edge of the bridge; the sensor nodes 220–235 form the second row and are located in the middle of the bridge; the sensor nodes 320–335 form the third row of sensor arrays. The sensor data used for the numerical analysis are chosen from the forced vibration tests with vertical directionality. The length of the sensor data time series is 5000. The shifted distances are measured by the centroids of the normal feature vectors and the damage pattern 6 feature vectors. From Figure 22, we can see that the closer the sensor nodes to the damage location (pier 3), the larger the shifted distance from pattern 6 feature vectors to the normal feature vectors. This result shows the potential of using pattern recognition approach for damage localization analysis. 

### 4.7. The Effects of Time Series Length on Computing Time

**Figure 23:**
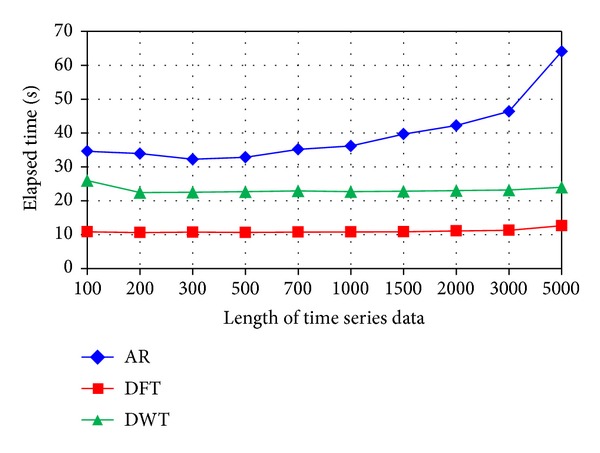
The impact of the time series length on the computing time for different feature extraction methods.

To investigate the impact of the time series length on computing time, simulation tests were performed using different feature extraction methods and with various time series lengths. The evaluation tests were conducted using a Dell computer with Intel Core2 Quad 2.4 GHz CPU and 4 GB of RAM.  Figure 23 shows the computing time for three feature extraction methods when the length of the time series changes. For the DFT- and DWT-based feature extraction methods, the length of the time series does not have significant impact on the computing time. For the AR-based feature extraction method, the computing time increases when the length of the time series increases.

## 5. Conclusions

This paper presents the research results of three feature extraction methods: autoregressive model, discrete Fourier transform, and discrete wavelet transform, for structural damage pattern recognition. The performance of a number of dissimilarity measures for feature extraction and pattern recognition is also investigated. The test data for evaluating the performance of feature extraction methods and dissimilarity measures are chosen from the Z24 bridge test. The Z24 bridge test data include the progressive damage data of the same type but varying levels as well as radically distinct damage modalities. These features of the damage data allow us to evaluate the performance of feature extraction methods and dissimilarity measures for different damage modalities and different levels of damage severity. The comparison results show that the combination of AR-based feature extraction and the Mahalanobis distance presents better performance compared with other feature extraction methods and dissimilarity measures. Although the computing time of AR-based feature extraction will increase when the length of a time series is longer than 1,000 data points, this will not impede the application of AR method. The reason is that the success rate of AR-based pattern recognition is already high when the length of a time series is 700 data points for both scenarios 1 and 2. The success rate does not improve too much when the length is further increased. In addition to feature extraction and pattern recognition, the feasibility of using pattern recognition approach for damage localization analysis is also studied in this paper. The simulation result shows that the closer the sensor nodes to the damage location, the larger the distances of damage feature vectors shift from the normal pattern feature vectors. 

## Figures and Tables

**Table 1:** Description of progressive damage tests.

Test	Description
Pattern 1	No damage (missing/corrupted data)
Pattern 2	No damage, pier hinge added (baseline)
Pattern 3	Pier 3 settlement: 20 mm
Pattern 4	Pier 3 settlement: 40 mm
Pattern 5	Pier 3 settlement: 80 mm
Pattern 6	Pier 3 settlement: 95 mm
Pattern 7	Pier 3 foundation tilt
Pattern 8	No damage, pier 3 restored
Pattern 9	Concrete spalling: 12 m^2^
Pattern 10	Concrete spalling: 24 m^2^
Pattern 11	Landslide at abutment
Pattern 12	Concrete hinge failure
Pattern 13	Anchor head failure (2)
Pattern 14	Anchor head failure (4)
Pattern 15	Tendon wire failure (54/2)
Pattern 16	Tendon wire failure (100/4)
